# Ubiquitination is involved in secondary growth, not initial formation of polyglutamine protein aggregates in *C. elegans*

**DOI:** 10.1186/1471-2121-13-10

**Published:** 2012-04-11

**Authors:** Gregory A Skibinski, Lynn Boyd

**Affiliations:** 1 Department of Biological Sciences, University of Alabama in Huntsville, Huntsville AL 35899, USA

## Abstract

**Background:**

Protein misfolding and subsequent aggregation are hallmarks of several human diseases. The cell has a variety of mechanisms for coping with misfolded protein stress, including ubiquitin-mediated protein degradation. In fact, the presence of ubiquitin at protein aggregates is a common feature of protein misfolding diseases. Ubiquitin conjugating enzymes (UBCs) are part of the cascade of enzymes responsible for the regulated attachment of ubiquitin to protein substrates. The specific UBC used during ubiquitination can determine the type of polyubiquitin chain linkage, which in turn plays an important role in determining the fate of the ubiquitinated protein. Thus, UBCs may serve an important role in the cellular response to misfolded proteins and the fate of protein aggregates.

**Results:**

The Q82 strain of *C. elegans *harbors a transgene encoding an aggregation prone tract of 82 glutamine residues fused to green fluorescent protein (Q82::GFP) that is expressed in the body wall muscle. When measured with time-lapse microscopy in young larvae, the initial formation of individual Q82::GFP aggregates occurs in approximately 58 minutes. This process is largely unaffected by a mutation in the *C. elegans *E1 ubiquitin activating enzyme. RNAi of *ubc-22*, a nematode homolog of E2-25K, resulted in higher pre-aggregation levels of Q82::GFP and a faster initial aggregation rate relative to control. Knockdown of *ubc-1 *(RAD6 homolog), *ubc-13*, and *uev-1 *did not affect the kinetics of initial aggregation. However, RNAi of *ubc-13 *decreases the rate of secondary growth of the aggregate. This result is consistent with previous findings that aggregates in young adult worms are smaller after *ubc-13 *RNAi. mCherry::ubiquitin becomes localized to Q82::GFP aggregates during the fourth larval (L4) stage of life, a time point long after most aggregates have formed. FLIP and FRAP analysis indicate that mCherry::ubiquitin is considerably more mobile than Q82::GFP within aggregates.

**Conclusions:**

These data indicate that initial formation of Q82::GFP aggregates in *C. elegans *is not directly dependent on ubiquitination, but is more likely a spontaneous process driven by biophysical properties in the cytosol such as the concentration of the aggregating species. The effect of ubiquitination appears to be most significant in later, secondary aggregate growth.

## Background

A number of diseases are known as protein misfolding disorders, or "conformational" diseases due to their association with protein misfolding and aggregation [1]. The most well-known of these are neurodegenerative diseases and include Alzheimer's, Parkinson's, and Huntington's disease. Other conformational diseases include cystic fibrosis and the muscle-wasting disease inclusion body myositis. The proteins underlying these diseases vary in sequence and structure, and the exact cause of the aberrant folding cannot always be attributed to specific mutations. However, these diseases are commonly characterized histologically by the presence of insoluble intracellular protein aggregates. These aggregates generally contain the misfolded species along with a variety of other proteins. One common hallmark of aggregates in conformational diseases is the presence of ubiquitin [2-6]. While it is not entirely clear whether the ubiquitin in aggregates has been conjugated to the misfolded protein itself, to other aggregating species, or aggregates as a free monomer, its presence indicates a potential role for ubiquitin in the aggregation process or in the cellular response to aggregation.

Ubiquitination is an important post-translational modification marked by regulated covalent attachment of the 8-kDa protein ubiquitin to specific cellular protein targets [7]. Through the action of the E1-E2-E3 series of enzymes, a high degree of substrate specificity is attained thus affording tight spatial and temporal control of a variety of cellular processes. Membrane protein transport [8], DNA damage repair [9], and histone regulation [10] are all known to be directly regulated by ubiquitination. The most well known fate of ubiquitinated protein substrates is degradation by the 26 S proteasome. Targeting of substrate proteins to the proteasome for degradation is mediated by the sequential attachment of ubiquitin to form a polyubiquitin chain of at least 4 ubiquitins, linked serially via isopeptide bonds between the C-terminal glycine of one ubiquitin to the ε-amino group of lysine 48 (K48) on the next [11]. Thus, the ubiquitin-proteasome system allows for specific degradation of protein targets and regulates cellular processes by controlling the half-life of proteins in pathways such as the cell cycle [12]. In addition to regulating cellular pathways, the ubiquitin-proteasome system is also important in the degradation of misfolded or damaged proteins, as part of protein quality control (PQC) system. During misfolded protein stress, cellular levels of ubiquitinated proteins increase significantly [13]. Polyubiquitin chains formed by attachment of successive ubiquitins through lysine 63 (K63) have been found to mediate endocytosis [14], NFκB function [15], and trafficking of proteins to form perinuclear aggregates known as aggresomes [16].

The E1 enzyme for ubiquitination in *C. elegans is *UBA-1. There is a single E1 for ubiquitin in C. elegans and 26 E2s, the UBCs or UEVs [17,18]. A UBC accepts ubiquitin from the E1, then directly transfers ubiquitin to either the target substrate or to a cysteine residue of a HECT domain E3. The E3 ubiquitin ligases are important in substrate recognition. A single UBC can interact with multiple E3s, and a single E3 can likely interact with multiple UBCs. Specific UBCs can play a role in determining the type of polyubiquitin chain formed on a target substrate. For example, the *Saccharomyces cerevisiae *Ubc13p, in conjunction with Mms2p, catalyzes the formation of K63-linked polyubiquitin chains [19], while yeast Ubc1p catalyzes the formation of K48-linked chains [20]. In addition, it appears that some chain-extending UBCs cannot initiate polyubiquitination without a previously conjugated acceptor ubiquitin [21]. Thus, an E3's choice of interacting UBC can determine the type of ubiquitination on the target, and its fate.

Proteins containing pathogenic polyglutamine expansions, such as those observed in Huntington's disease, spinal bulbar muscular atrophy, and spinocerebellar ataxia are particularly prone to aggregation and formation of ubiquitin-positive inclusions [22]. In vivo and in vitro studies on the misfolding and aggregation of polyglutamine proteins have suggested a model in which one or more soluble, metastable polyglutamine monomers form a critical nucleus that is prone to oligomerisations. An autocatalytic feedback loop involving the misfolded monomers and oligomers promotes the transition of more monomers to the aggregation-prone conformation, accelerating the oligomer formation [23].

There is ongoing debate regarding the toxicity of intracellular protein aggregates. Some evidence suggests that the aggregates may sequester non-pathological cellular proteins and may lead to loss-of-function phenotypes for these proteins [24,25]. Proteins containing polyglutamine tracts are particularly prone to co-aggregation with other polyglutamine proteins [26]. In cell culture models, expression of protein aggregates can cause impairment of the ubiquitin-proteasome system [27,28]. Later studies supported a model where the early or intermediate forms of protein aggregates caused proteasomal impairment, which was relieved by inclusion body formation [29-31]. Still, other evidence suggests that the large aggregates may be neutral or cytoprotective for the cell [32-36].

The formation of aggregates may aid the cell's proteolytic mechanisms in ridding the cell of the misfolded protein. Chen and associates found that some nuclear inclusions co-localize with areas of proteasomal proteolysis [37]. Localization of proteasomal machinery has been shown with polyglutamine-containing aggregates in a p62-dependent manner [38]. Cytosolic aggregates may be subject to degradation by autophagy [39] as well as by the ubiquitin-proteasome system [40].

In some cases, it appears that the cell actively transports misfolded proteins into juxtanuclear, pericentriolar, vimentin-caged inclusions termed "aggresomes", a process that involves ubiquitination [41]. This may reflect an adaptation by cells that serves to accumulate damaged proteins in a single location, allowing for more efficient degradation by autophagy. The transport to aggresomes along microtubules is facilitated by K63-linked polyubiquitin chains. p62 may also be involved in directing aggregates to non-proteasomal protein degradation by autophagy [38]. K63-polyubiquitinated proteins are transported by dynein-dynactin complexes via the adapter histone deacetylase 6 (HDAC6) [42]. Formation of aggresomes may be protective, as experiments in which formation of these structures is inhibited results in increased cytotoxicity to cultured cells [43]. In addition, aggresomes appear to protect cells expressing the Parkinson's disease-associated proteins alpha-synuclein and synphilin-1 [44].

Protein aggregation appears to be associated with non-disease biology as well. A recent study found that general protein insolubility increases with age in *C. elegans *[45]. In addition, protein aggregation may play an important role in the immune system. Dendritic cell aggresome-like-induced structures (DALIS), which contain ubiquitinated proteins, have been hypothesized to be involved in temporary storage of antigens during maturation of dendritic cells [46].

It has been beneficial to develop animal, cell, and in vitro systems for studying protein aggregation. Morimoto et al., have developed a transgenic strain of *C. elegans (*henceforth referred to as Q82) that expresses an aggregation prone stretch of 82 glutamines fused to GFP (Q82::GFP) in the body wall muscle cells [47]. The polyglutamine reporter protein aggregates into distinct puncta that recapitulate many of the features of disease aggregates, including insolubility [47], interactions with chaperones [48], and positive staining for ubiquitin [49]. In a previous RNAi screen, our lab demonstrated that RNAi knockdown of specific UBCs affects the size, number, and ubiquitin immunoreactivity of these aggregates in the Q82 strain [17]. Specifically, RNAi of *ubc-1, ubc-13*, and *uev-1 *resulted in significantly smaller aggregates that did not stain positively for ubiquitin or proteasome. RNAi of *ubc-2 or ubc-22 *resulted in larger aggregates that were fewer in number. Similar effects were seen after RNAi of human homologs of these genes in cultured HEK293 cells [17].

The current study expands upon those results by examining dynamics of aggregate formation in vivo. Time-lapse fluorescence microscopy of Q82::GFP reveals a biphasic nature to polyglutamine aggregation in *C. elegans*. Initial formation of microscopically visible aggregates occurs rapidly and is largely unaffected by knock down of UBCs. RNAi of *ubc-22 *resulted in higher levels of initial fluorescence. RNAi of *ubc-13 *impedes growth of aggregates during the secondary growth phase. Furthermore, we examined the dynamics of aggregate ubiquitination by use of a fluorescent mCherry::ubiquitin fusion protein and found that localization of this protein to the Q82::GFP aggregates is secondary to initial formation.

## Results

### Initial Q82::GFP aggregate formation occurs rapidly in L1 and L2-stage worms

**Figure 1:**
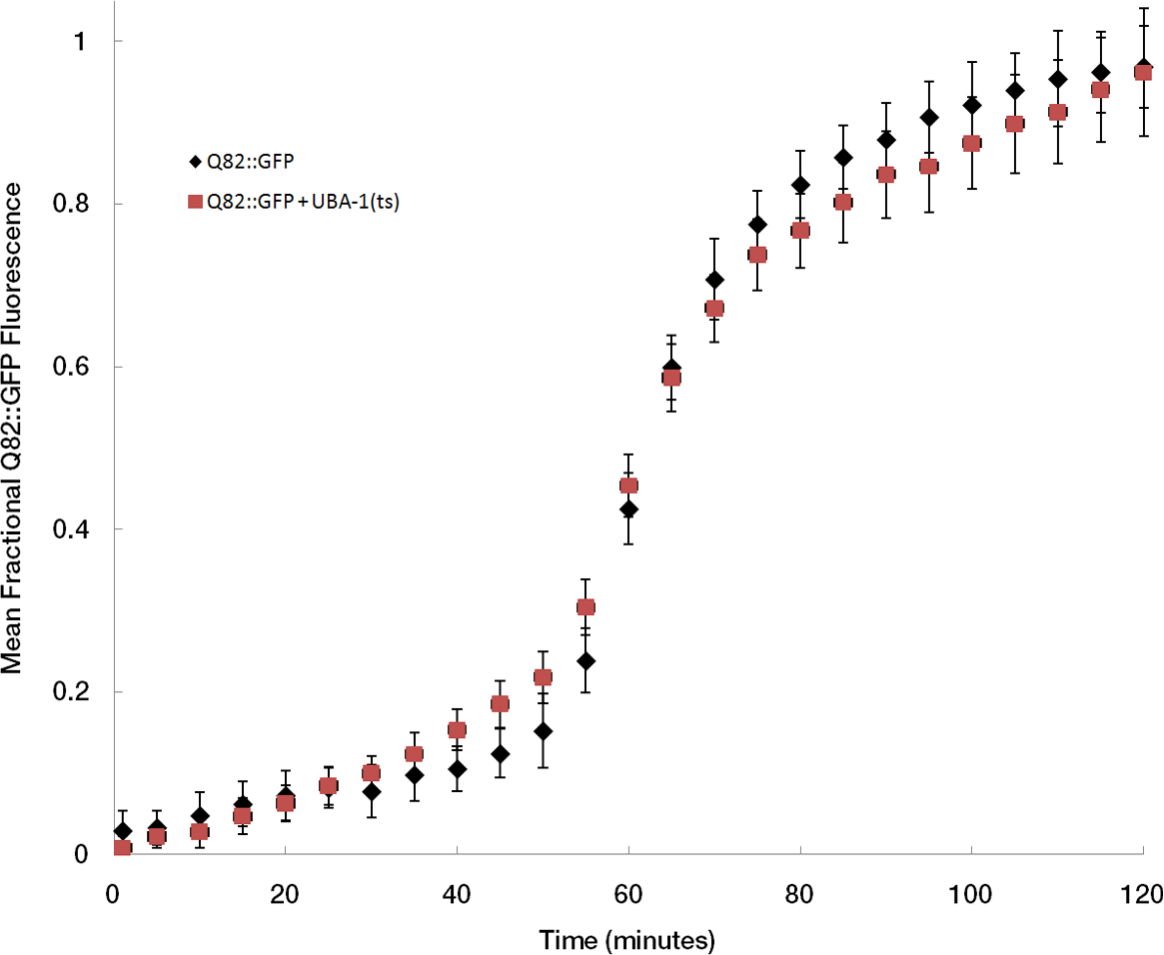
**Aggregation profile of Q82::GFP protein in *C. elegans***. *C. elegans *larvae expressing a Q82:GFP transgene in a wild type (black diamonds) or in a *uba-1 *mutant (red squares) were imaged using a microscope with a 10X objective lens at a rate of 1 frame per minute. The sum of the pixel intensity in a square region of the image sequence in which an aggregate formed was measured over time. Plots of individual formation events were aligned along the time axis so that the frame at which the aggregation rate is highest occurs at 60 minutes. For each individual aggregate formation, the fraction of the peak intensity was calculated at each time point. This chart shows the mean (± SEM) fractional aggregate intensity at each time point for a total of 58 aggregate formations in 5 different time-lapse experiments for the Q82 strain in the wild type background and 30 aggregate formations in 3 experiments for the Q82 in the *uba-1(ts) *background.

The transgenic Q82 strain of *C. elegans *expresses a fusion protein that consists of 82 glutamine residues fused to GFP, under control of the *unc-54 *promoter for expression in the body wall muscle cells. Aggregates form throughout the animal's development to adulthood [47]. We have employed this model to examine the early formation of aggregates, and the role of ubiquitination in this process. L1- and L2-stage worms readily form distinct puncta of Q82::GFP that appear concurrently with the disappearance of diffuse, putatively soluble fluorescent material (Additional files 1 and 2 Figure: Videos 1 and 2). This process of initial aggregate formation occurs rapidly, with the time for aggregation to occur (defined as the time taken for total fluorescence to increase from 10% to 90% of maximum) being 58.1 *± *21.5 minutes. Initial formation is represented by a sigmoidal curve (Figure 1) when plotted as a function of time. This is followed by secondary growth, in which fluorescence increases at a slower rate. Aggregates forming during time-lapse observation are similar in size to those that were formed prior to microscopic observation. They consists of largely immobile Q82::GFP, as they do not show fluorescence recovery after photobleaching (G.S., data not shown).

In order to examine the potential role of ubiquitination in the aggregation process, the Q82::GFP transgene was crossed into a strain harboring a temperature sensitive mutation in the *uba-1 *gene which is the single gene encoding the ubiquitin E1 activating enzyme in *C. elegans*. It has previously been shown via immunoblots with anti-ubiquitin antibodies that overall ubiquitination is dramatically reduced (> 90% reduction in ubiquitin conjugates) under nonpermissive conditions (25°C) in this mutant [50]. Figure 1 shows that in the time-lapse assay, under nonpermissive conditions, reduction of UBA-1 activity does not change the kinetics of single formation events, as it does not significantly affect the sigmoidal shape of the curve, the maximum rate of formation, or the time taken for an aggregate to form. This result suggests that the process of initial aggregate formation may not be directly dependent on ubiquitination.

### RNAi knockdown of UBCs has limited effect on the initial aggregate growth phase

**Figure 2:**
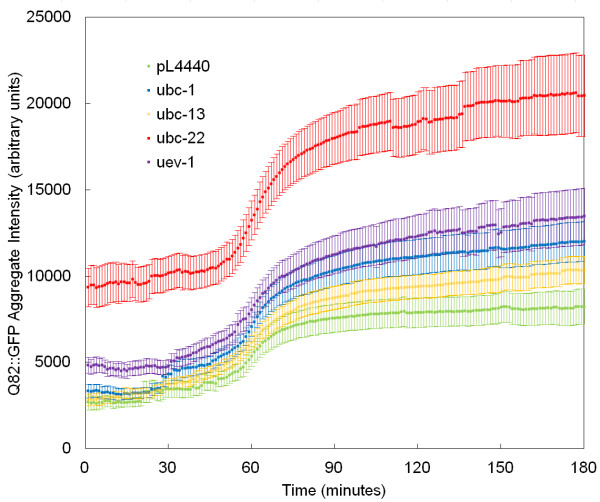
**RNAi knockdown of UBCs affects the cellular level and aggregation rate of Q82::GFP fusion protein**. *C. elegans *expressing a Q82:GFP transgene were fed bacterial clones expressing dsRNA against the indicated genes or the empty pL4440 vector as a control, beginning at the L2 stage. The progeny of these worms were imaged at a rate of 1 frame per minute. Pixel intensity in a square region of the image sequence in which an aggregate formed was measured over time. Plots of individual formation events were aligned along the time axis so that the frame at which the aggregation rate is highest occurs at 60 minutes. This chart shows the mean (± SEM) aggregate intensity at each time point for each RNAi treatment. At least 50 aggregates were analyzed for each RNAi treatment. See Table 1 for calculation of aggregation rates.

We have previously shown that RNAi knockdown of specific UBCs can affect the size and number of aggregates in worms at an age of 48 hours [17]. Specifically, the most dramatic phenotypes were seen with *ubc-1, -2, -13, -22*, and *uev-1*. Therefore, these UBCs were chosen for further analysis. For RNAi-mediated knockdown, the identical RNAi feeding strains and protocols were used that have been shown to reduce RNA to barely detectable levels [17]. Time-lapse microscopy was used to observe initial formation of aggregates in UBC knock down worms. RNAi treatment was started in L2 worms, and the L1 progeny of those worms were used for time-lapse imaging. Fluorescence measurements of time-lapse image series were made by recording the total intensity of all pixels over time in a square region framing the boundaries of the final aggregate. After performing multiple experiments, aggregation data were pooled by treatment group, aligned for the coincidence of aggregate formation, averaged at each time point, and plotted to form composite curves (Figure 2). Notably, RNAi of *ubc-22*, and, to a lesser extent, *uev-1*, resulted in an increase in initial levels of Q82::GFP, and an increase in the rate of initial aggregate formation. To compare the effects of the RNAi treatments on the rate of aggregation, a linear regression was performed on composite curves at the period of initial rapid aggregation (minutes 58-62) These data are shown in Table 1. The table shows that almost all UBCs had an effect on initial fluorescence levels. This may be due to a general disruption in the UPS by disruption of key components in that pathway. *ubc-13 *was the only UBC to show no significant effect on initial fluorescence level. In general, higher initial levels of Q82::GFP are associated with higher rates of aggregation. This correlation suggests that once an aggregate is initially seeded, its formation is largely diffusion-limited and dependent on concentration, rather than active cellular pathways.

**Table 1:** Initial fluorescence levels and aggregation rates of Q82::GFP fusion protein

RNAi treatment	Initial fluorescence level prior to aggregation (± SEM)	Aggregation rate of initial formation (change in pixel intensity minutes^-1^)
pL4440		
(empty vector)	2712 ± 452	208.1
*ubc-1*	3335 ± 394	286.2
*ubc-13*	2827 ± 347	177.9
*ubc-22*	9349 ± 1124	344.9
*uev-1*	4847 ± 450	255.8

### RNAi knockdown of UBCs influences the secondary aggregate growth phase

**Figure 3:**
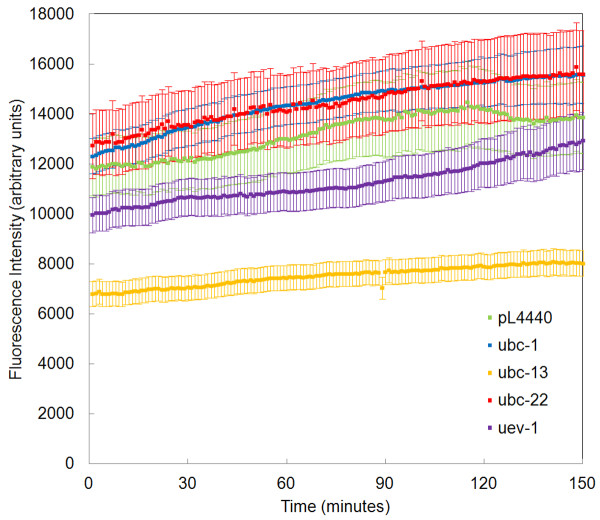
**RNAi knockdown of UBCs alters the secondary growth of aggregates**. *C. elegans *expressing a Q82::GFP transgene were fed bacterial clones expressing dsRNA against the indicated genes or the empty pL4440 vector as a control, beginning at the L2 stage. The progeny of these worms, belonging to the same population as the worms analyzed in Figure 2, were imaged using a microscope with a 10X objective lens at a rate of 1 frame per minute. Aggregates that had formed prior to mounting on the slide were located using the automatic object tracking feature of Image Pro Plus 6.1, which uses an intensity threshold to define the borders of the aggregates. The sum pixel intensity within each automatically defined region was measured over time, omitting objects that formed during the observation period. The Y axis represents the mean pixel intensity for all aggregates, plotted by gene. At least 50 aggregates were analyzed for each RNAi treatment. See Table 2 for calculation of aggregation rates.

In the *C. elegans *Q82 model, aggregates in adult worms are much larger than those seen in young larvae. The data Figures 1 and 2 show that initially aggregates form quickly, but that after initial formation, aggregates continue to grow at a slower rate. Since RNAi of UBCs affects aggregate size, we wanted to investigate whether the UBCs might affect this secondary growth phase. Thus, from the same population of worms in which we observed aggregate formation, we recorded the growth of aggregates that had formed prior to observation under the microscope (Figure 3). Table 2 summarizes the growth rates of these aggregates, as determined by a linear regression performed on the data from Figure 3. In this case initial fluorescence level indicates the level of fluorescence in the existing aggregate at the time of observation during the L1 larval period. Initial fluorescence of aggregates was higher in worms subjected to RNAi of *ubc-22*, consistent with our previous findings [17]. In addition, *ubc-*22 and *ubc-1 *RNAi aggregates grew at rates faster than the control (Table 2). Aggregates in worms treated with *ubc-13 *and *uev-1 *RNAi showed smaller aggregates, consistent with results from our previous study [17]. In addition, secondary growth was markedly reduced upon RNAi of *ubc-13 *(Table 2). Since homologs of *ubc-13 *and *uev-1 *are known to dimerize and to catalyze the formation of K63-linked polyubiquitin chains [9,19], it is possible that the reduced secondary growth rate may be related to reduced levels of K63 ubiquitination.

**Table 2:** Initial fluorescence levels and growth rates of pre-formed aggregates

RNAi treatment	Initial fluorescence level	Growth rate of pre-formed aggregates (change in pixel intensity · minutes^-1^)
pL4440		
(empty vector)	11761 ± 1131	17.52
*ubc-1*	12224 ± 1078	21.17
*ubc-13*	6781 ± 510	8.95
*ubc-22*	12546 ± 1287	19.19
*uev-1*	9927 ± 706	17.40

### Aggregates of Q82::GFP contain both K48- and K63-linked polyubiquitin chains

**Figure 4:**
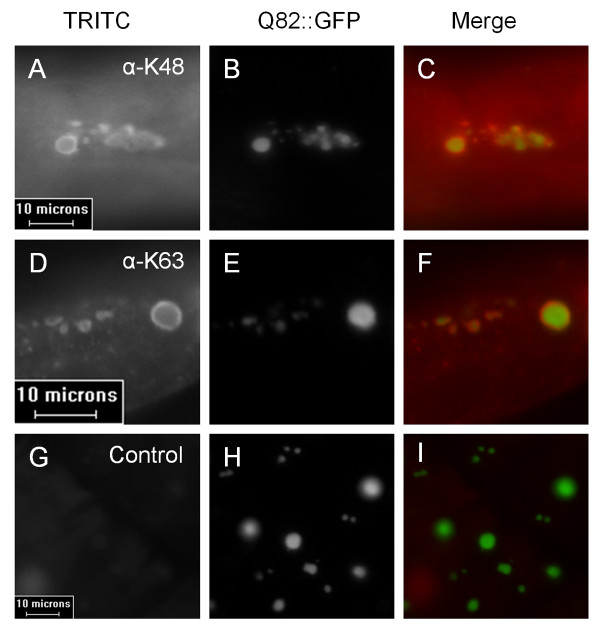
**Q82::GFP aggregates contain both K48- and K63-linked polyubiquitin chains**. Synchronized adult worms were fixed and probed with antibodies specific for K48 or K63 polyubiquitin chains. Secondary antibody was conjugated to TRITC (**A**, **D**, and **G**). Aggregates were visualized via GFP fluorescence (**B**, **E**, **H**). Control samples (**G**) received the same treatment, without primary antibody. Merged images (**C**, **F**) show colocalization of K48- or K63-linked polyubiquitin chains to aggregates, but not when the primary antibody is omitted (**I**).

Q82::GFP aggregates in the Q82 strain stain positive for ubiquitin using a pan-ubiquitin primary antibody, similar to intracellular aggregates in many diseases [17]. Since the linkage type of a polyubiquitin chain attached to a substrate is important in determining that protein's fate, examining the types of polyubiquitin chains present in the aggregates may provide clues as to the role of ubiquitin in aggregate formation. Using antibodies specific to K48- or K63-linked polyubiquitin chains in an immunofluorescence assay, we found that the Q82::GFP aggregates in adult worms stain positive for both linkage types (Figure 4). Both large, spherical aggregates and the smaller, more granular aggregates appear to contain both polyubiquitin chain types. Specificity of the antibodies was supported by the observation that only the K48 antibody stains interphase nuclei while the K63 antibody stains diakinetic chromosomes in meiotic cells (G.S., data not shown).

### Higher levels of ubiquitin colocalization are seen in L4 stage worms

**Figure 5:**
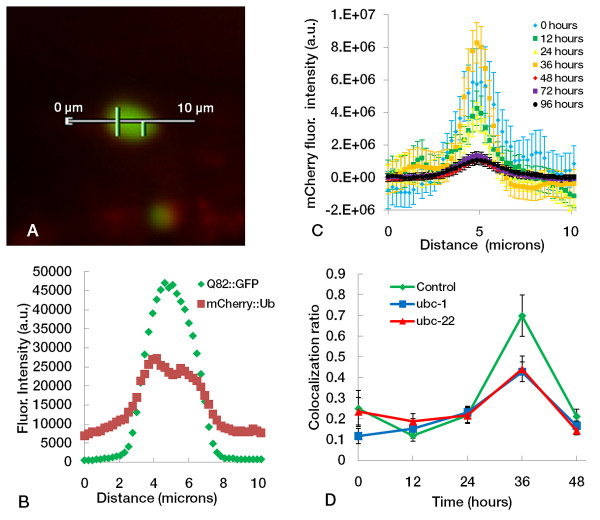
**mCherry::ubiquitin colocalization to Q82::GFP aggregates**. Worms co-expressing an mCherry::ubiquitin with Q82::GFP were exposed to RNAi of *ubc-22, ubc-1*, or pL4440 control. A typical control aggregate is shown in (**A**) with a 10.16-micron line (45 pixels) measuring a cross-section of fluorescence intensity. Data for this line profile is shown in (**B**). (**C**) Averaged line profile data for mCherry::ubiquitin fluorescence of Q82::GFP aggregates at multiple time points. Higher levels of colocalization of mCherry::ubiqutin occur at an age of 36 hours. Peaks represent fluorescence values in the aggregates while the peripheral points represent intracellular fluorescence. Values are the mean (± SEM) of all values at that distance coordinate for aggregates at the given time point. All values have been normalized with respect to exposure time and background levels of fluorescence. (**D**) Time course of mCherry::ubiquitin colocalization to Q82::GFP aggregates shows the spike in ubiquitin colocalization at 36 hours, which is diminished by RNAi of *ubc-1 *or *ubc-22*. Data shown are the mean (± SEM) ratio of mCherry fluorescence to GFP fluorescence from line profile measurements of composite images for multiple aggregates at each time point.

A strain expressing mCherry::ubiquitin with Q82::GFP in muscle cells (hereby referred to as Q82 + Ub) was used to assess the dynamics of ubiquitin localization to aggregates. A time course of mCherry::ubiquitin colocalization was performed, in which worms were imaged every 12 hours and line profile measurements of individual aggregates were taken to evaluate mCherry::ubiquitin colocalization to Q82::GFP aggregates. Our results show a spike in mCherry::ubiquitin colocalization in aggregates at 36 hours (Figure 5C, D). In the same experiment, groups exposed to RNAi of *ubc-1 *or *ubc-22 *show reduced colocalization of mCherry::ubiquitin to the polyglutamine aggregates (Figure 5D) at 36 hours.

The results indicate that ubiquitin localization to polyglutamine aggregates varies with developmental stage. This finding is consistent with other reports suggesting that the protein quality control pathways are not constant throughout all periods of the life cycle. Interestingly, the ratio of mCherry to GFP fluorescence seems to decrease from 36 hours to 48 hours (Figure 5D). As Q82::GFP aggregates increase in size during this time, the decrease in mCherry::ubiquitin colocalization may be the result of the activity of deubiquitinating enzymes (DUBs).

### Photobleaching experiments reveal mobility of mCherry::ubiquitin within Q82::GFP aggregates

**Figure 6:**
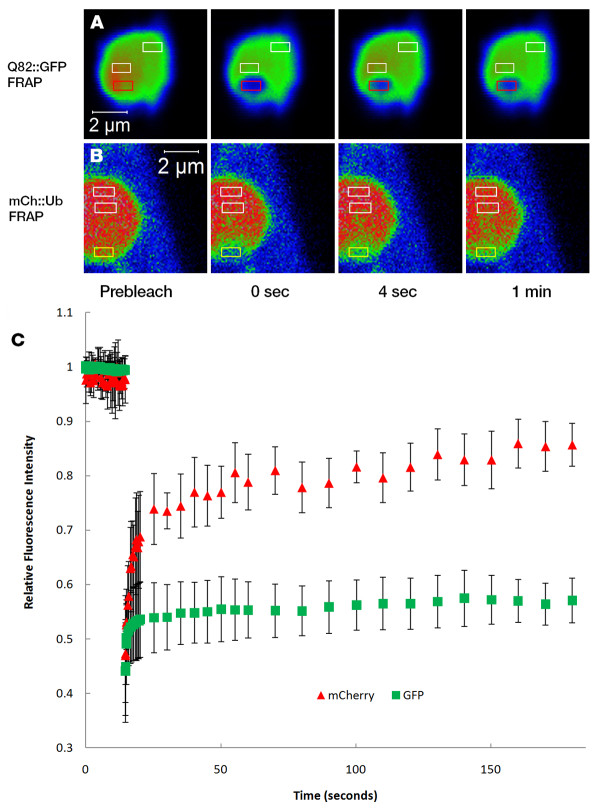
**FRAP analysis of mCherry::ubiquitin and Q82::GFP in polyglutamine aggregates reveals differential mobility of ubiquitin and polyglutamine proteins within aggregates**. Worms co-expressing an mCherry::ubiquitin fusion protein with Q82::GFP were subjected to FRAP using a confocal laser scanning microscope system. Fluorescence intensity is indicated by a heat map of either Q82::GFP (**A**) or mCherry::ubiquitin (**B**). Red or yellow rectangles indicate regions to where bleaching was directed. White rectangles indicate regions that were used to control for acquisition photobleaching. Measurements of fluorescence recovery were taken every 0.1 s for 3 minutes. Quantitative analysis (**C**) of fluorescence recovery in bleached regions indicates higher overall mobility of mCherry::ubiquitin fusion protein when compared to Q82::GFP protein. Data plotted are the mean ± SEM.

Ubiquitin within aggregates may be attached to the primary aggregating protein, attached to other proteins that coaggregate, or associated as a free monomer. The mobility of the protein may provide insights into its conjugation state. FRAP and FLIP experiments were performed in order to examine the mobility of mCherry::ubiquitin within the aggregates of Q82::GFP. In the FRAP experiments, mCherry or GFP was bleached within a region of the Q82::GFP aggregates in adult worms and recovery was observed (Figure 6A, B). Q82::GFP showed a slight recovery (Figure 6C) with a mobile fraction of 23.3 ± 9.2. The mCherry::ubiquitin fusion protein showed a higher degree of recovery, with a mobile fraction of 70.8 ± 17.0. This result indicates that while the polyglutamine protein, Q82::GFP, is highly immobile within aggregates, ubiquitin shows a greater rate of diffusion. The slow, continued increase in mCherry recovery after the initial rapid recovery may indicate the continued accumulation into aggregates of mCherry::ubiquitin or substrates to which it is attached.

**Figure 7:**
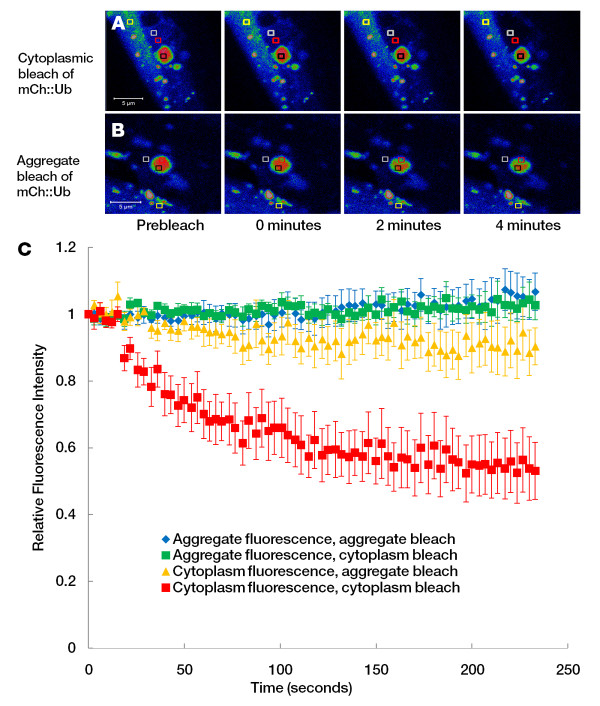
**FLIP analysis of mCherry::ubiquitin and Q82::GFP**. Worms co-expressing an mCherry::ubiquitin fusion protein with Q82::GFP were subjected to FLIP analysis of the mCherry::ubiquitin protein to study mobility of ubiquitin. Fluorescence intensity is indicated by a heat map of mCherry::ubiquitin prior to bleaching and at various times after commencement of repeated bleach pulses. Red squares indicate regions where bleach pulses were directed, black and white squares indicate regions that were quantitatively analyzed for fluorescence loss, and yellow squares indicate regions in non-bleached cells that were used to control for acquisition photobleaching. Separate experiments were carried out in which bleaching was directed to the cytoplasm (**A**) or the aggregate of Q82::GFP (**B**). A quantitative analysis (**C**) was carried out to analyze fluorescence loss over time. Results indicate no loss of mCherry fluorescence in aggregates when bleaching was directed to either a separate region within the aggregate itself (blue diamonds) or an area in the cytoplasm. The loss of fluorescence in the cytoplasm when a region within the cytoplasm was bleached indicates the effectiveness of the bleaching protocol (red squares), while the limited loss of fluorescence in the cytoplasm when a region within the aggregate was bleached indicates the limited access of mCherry::ubiquitin to the Q82::GFP aggregates.

To further investigate the mobility of mCherry::ubiquitin within Q82::GFP aggregates, fluorescence loss in photobleaching (FLIP) was used. mCherry was continuously bleached in a region either within the Q82::GFP aggregate or in the cytoplasm of a cell expressing the two fusion proteins. Loss of fluorescence in either a separate region within the aggregate or in the cytoplasm was monitored to examine mobility of the fluorescence material (Figure 7A, B). Directing bleach pulses to either the cytoplasm or the aggregate itself did not result in loss of fluorescence within the aggregate, indicating mCherry::ubiquitin is sequestered within aggregates. Bleaching within the cytoplasm reduced cytoplasmic mCherry fluorescence, indicating the effectiveness of the bleaching protocol and the mobility of mCherry::ubiquitin within the cytoplasm (Figure 7C). These results support the notion that the mCherry::ubiquitin is sequestered within the Q82::GFP aggregates, but is not itself in an aggregated, immobile configuration.

## Discussion

The presence of ubiquitin within protein aggregates is a common feature in multiple diseases. In addition, many of these diseases are age-related and may be associated with impairment of protein quality control. Thus, it is important to investigate the role that the ubiquitination machinery plays in the process of aggregation. In this study we examined the early formation of intracellular polyglutamine aggregates, and found that ubiquitin conjugating enzymes do not play direct roles in initial aggregation, but likely are responsible for ubiquitinating substrates that are recruited to aggregates later.

### Initial Q82::GFP aggregation occurs rapidly and is not directly dependent on ubiquitination

Initial formation of Q82::GFP aggregates in *C. elegans *muscle cells completes in approximately 1 hour. This seems to be a rapid process, when considered in the context of the slow, age associated onset of many polyglutamine diseases. Aggregation rate was correlated with Q82::GFP concentration immediately prior to aggregate formation. A dependence of aggregation rate on concentration is consistent with a model in which the initial aggregation is a spontaneous, entropy-driven process that is dependent on biophysical parameters such as temperature and diffusion coefficient. Interestingly, the aggregation curves for Q82::GFP are sigmoidal, which is suggestive of a model of nucleation followed by autocatalytic growth. Indeed, in vitro experiments lacking ubiquitination machinery produced aggregation curves that fit a sigmoidal curve [51]. It should be noted that the standard fluorescence microscopy techniques used in this study are unable to detect the formation of smaller, subresolution Q82::GFP oligomers and protofibrils. Such smaller species may be forming during the "lag time" prior to rapid aggregation, as seen in vitro [52].

The observation that formation time does not change significantly in worms in which ubiquitination has been impaired due to a temperature sensitive mutation in *uba-1 *further suggests that ubiquitination is not required for initial aggregation in this model. In support of this model, only very low levels of mCherry::ubiquitin are seen colocalizing to Q82::GFP aggregates in young worms, the age in which aggregates begin appearing (within 24 hours of hatching). Similar results are seen in the ubiquitination of the Parkinson's Disease protein alpha-synuclein. In transfected cells, mutant alpha-synuclein that cannot be ubiquitinated can still form aggregates, though the propensity for aggregation increases when it is mono- or di-ubiquitinated [53].

A higher level of initial fluorescence is seen after RNAi of *ubc-22 *and is associated with a higher rate of aggregation, both in the initial formation and the secondary growth phases. A higher level of Q82::GFP fluorescence may indicate a higher concentration of the fusion protein in the area where the aggregate forms. Thus, it is possible that UBC-22 normally functions in the degradation of Q82::GFP. Eliminating its function may allow Q82::GFP to accumulate, resulting in larger aggregates formed from the increased quantity of intracellular protein. E2-25K, the human homolog that is most closely related to UBC-22 (32% identity), has been shown to be involved in the formation of K48-linked polyubiquitin chains [54] and to interact with the polyglutamine-containing protein huntingtin [55]. Since K48 chains are associated with proteasomal degradation, this UBC may be involved in targeting aggregating proteins for degradation.

The role of UBC-22 may be in localized or restricted degradation of Q82:GFP since levels of GFP fluorescence levels measured in whole worms did not show differences between control and *ubc-22 *RNAi (G.S, data not shown). Thus, UBC-22 may have some role in preventing the accumulation of Q82::GFP in specific regions of the cell. Alternatively, UBC-22 may play a role in setting the threshold for initial aggregation. As cytosolic levels of Q82::GFP increase, molecular crowding can decrease the quality of the protein folding environment and increase the probability of polyglutamine proteins adopting an aggregation-prone conformation. RNAi of *ubc-22 *may increase the threshold concentration at which aggregates appear. A likely mechanism that allows levels of polyglutamine protein to increase locally without aggregating involves chaperones. It is possible that UBC-22 normally inhibits chaperone activities, such as causing the ubiquitination and degradation of chaperones.

UBC-22 is an atypical UBC in that the cysteine residue (C74) near the putative catalytic domain is positioned 12 residues N-terminal to its expected position. This positioning brings into question whether this UBC is truly catalytically active. UBC-22 also appears to lack some residues critical in binding ubiquitin in the UBC domain. In Ubc1, the yeast ortholog of UBC-22, docking analysis shows that residues L89-I91 and N119-P121 interact with ubiquitin when the C-terminus of ubiquitin is placed at the active site cysteine residue of Ubc1 [56]. UBC-22 lacks corresponding residues. The same analysis also revealed several residues (E117, D120, and A111) in Ubc1 that appear to form hydrogen bonds with ubiquitin. UBC-22 has closely corresponding residues (E101 and D110) for E117 and D120, but, unlike E2-25K, lacks a residue corresponding to A111. Interestingly, a yeast two-hybrid analysis showed that UBC-22 interacts with the RING finger E3 ligase NHL-1 [57]. Further experiments exploring the interaction of UBC-22 and NHL-1 and their effects on aggregation may provide insight into the role of UBC-22.

### Ubiquitin conjugating enzymes may affect aggregation during the secondary growth phase

After the initial aggregation phase, polyglutamine aggregates continue growth with a slower, secondary growth phase. This secondary rate of growth is reduced by RNAi of *ubc-13 *and to a lesser extent, *uev-1*. UBC-13 and UEV-1 have been shown to interact in a yeast two-hybrid experiment [58], S. Utarrala and L.B., unpublished data] and therefore may function as a dimer. In yeast, this dimer is capable of catalyzing the formation of K63-linked polyubiquitin chains. Since our current study shows that K63 linked chains are present at polyglutamine aggregates, it is possible that proteins associated with K63 ubiquitination may promote the secondary growth of polyglutamine protein aggregates. This mechanism may also explain the apparent disparity between the relative sizes at the end of the initial aggregate formation (Figure 2), and the beginning of the observation of mature aggregates (Figure 3). As initial formation was observed largely in L1 and L2 animals, and mature aggregates observed in Figure 3 were largely found in L2 and L3 animals, the secondary growth rates during the intervening period can account for the observed differences. Specifically, *ubc-1 *worms exhibit the highest secondary growth rate (Table 2) and these aggregates become comparable in size to the initially larger *ubc-22 *aggregates over time. Also, *ubc-13 *worms show the slowest secondary growth rate (Table 2) and they then represent the smallest aggregate population observed in the mature aggregate population (Figure 3). We propose reduced K63-linked ubiquitination as a possible explanation for the lack of growth seen in *ubc-13*, and, to a lesser extent, *uev-1 *RNAi-treated animals.

In other systems, it has been proposed that K63 ubiquitination of aggregating proteins leads to trafficking of oligomers to aggregation sites [42]. It is unknown if this same scenario exists in the *C. elegans *muscle. In mammals, the adapter protein p62 has been implicated in this trafficking process [38]. p62 contains a UBA domain that is capable of binding to K63-linked polyubiquitinated proteins, and has been shown to promote aggregation in vivo and in vitro. *C. elegans *possesses a protein, SEPA-1, which, like p62, can recruit specific proteins to the autophagy machinery for degradation. However, it is unknown if SEPA-1 can bind polyubiquitin chains [59]. An alternative hypothesis is that K63-linked polyubiquitin chains may compete with K48-linked chains for binding to the proteasome [60]. It is possible that the reduction of K63-linked polyubiquitination in the cell might allow for increased degradation of K48-linked polyubiquitinated proteins thus depleting the cell of aggregation-prone proteins and lead to smaller aggregates.

mCherry::ubiquitin localization to aggregates is highest in the early L4 larval stage. This agrees with a time course where major ubiquitination events occur after initial aggregate formation. This accumulation of ubiquitinated proteins may be related to general changes in protein homeostasis that occur in *C. elegans *aging [61]. Alternatively, the late localization of ubiquitin to the aggregates could be the result of increased proteasome inhibition caused by protein aggregates. The results of our study agree with observations by Stenoien, et al. using a cotransfection assay of polyQ-expanded ataxin fused to cyan fluorescent protein (CFP) and yellow fluorescent protein fused to ubiquitin. They reported that small aggregates of CFP-ataxin-84Q were typically not ubiquitinated, while many larger aggregates contained high levels of ubiquitin [62]. Treating the co-transfected cells with the proteasome inhibitor MG132 resulted in accumulation of YFP-ubiquitin into aggregates. In addition, there is some histological evidence to suggest that ubiquitination of aggregates succeeds their formation. Studies using huntingtin or ubiquitin-specific antibodies to examine the brains from individuals suffering from varying clinical grades of HD found that ubiquitin was not present in all aggregates, and was present in a higher percentage of aggregates in higher clinical grades of the disease [63]. Animal studies found that in a mouse model of the polyglutamine expansion disease SCA7, the appearance of ubiquitin-positive aggregates occurred no earlier than 12 weeks of age, which is up to 7 weeks later than the earliest detection of aggregates in certain tissue [64].

### Ubiquitin is mobile but sequestered within Q82::GFP aggregates

In this study, both K48 and K63 ubiquitin chains were detected in Q82::GFP aggregates. Similar to many previous reports, we observed that the antibody staining is strongest in the periphery of the aggregates. The peripheral staining has prompted the question of whether that pattern was a result of ubiquitin being restricted to the periphery or whether the antibodies might be unable to penetrate into the interior of the aggregate. In the Q82 + Ub strain, many aggregates also show higher mCherry fluorescence in the periphery (Figure 5A, B), supporting the idea that ubiquitin is at a higher concentration at the peripheral regions of the aggregate. A tempting explanation for this is that ubiquitination is occuring on aggregates after initial formation.

The FRAP results are also consistent with later deposition of ubiquitinated proteins. Our results, like previous FRAP studies of aggregated fluorescent fusion proteins [45,48,65], show that there is little to no mobility of the aggregating species. Some aggregate associated proteins are seen to be mobile or transiently associated with aggregates, including chaperones [48], CBP, proteasome subunits [62]. Similarly, the mCherry::ubiquitin protein is largely mobile. The differences in mobility between Q82::GFP and mCherry::ubiquitin indicate that ubiquitin is not simply conjugated onto the polyglutamine protein. It may be brought to aggregates via its association with other proteins or it may exist in aggregates as a ubiquitin monomer. Differences in the conjugation state of the mCherry::Ub may also explain the biphasic nature of the FRAP curve for this protein. The rapid, initial recovery may be due to free mCherry::Ub diffusing into the measurement area, while the slower, secondary recovery may be occurring as ubiquitin chains or larger ubiquitinated proteins diffuse into this space. Alternatively, different topologies of mono- or polyubiquitin moieties may have different binding modes with respect to ubiquitin receptors sequestered within the aggregate, thus providing varying diffusion kinetics within this environment.

Interestingly, examination of the Q82::GFP aggregates using FLIP shows that mCherry::ubiquitin within the aggregates does not exchange significantly with the cytoplasm. This sequestration of ubiquitin into aggregates may indicate a high concentration of ubiquitin receptors within the aggregate. In accordance with this, cell based assays using polyQ-expanded ataxin found that aggregates of this protein contain ubiquitin binding structures including PUB motifs, ubiquitin-interacting motifs (UIMs), and ubiquitin-associated (UBA) domains [66]. Ubiquitinated proteins may be recruited to aggregates via binding to these domains. Of course, this prompts the question of how these ubiquitin binding proteins are recruited to aggregates. The proteasome, which contains both chaperone-like subunits and ubiquitin binding domains [67], may be the source of ubiquitin attracting activity in aggregates. Proteasomes may initially localize to aggregates via affinity for misfolded proteins. If proteasomes become engaged in failed attempts to unfold and degrade polyglutamine fibrils, this may lead to the accumulation of ubiquitinated substrates in the cytoplasm. Finally, accumulation of proteasomes at aggregates may explain the secondary accumulation of ubiquitin to the aggregates in our model. In support of this, our previous RNAi screen indicated that RNAi treatments that significantly reduce the size of aggregates also eliminated ubiquitin immunoreactivity in the aggregates, and RNAi of ubiquitin itself resulted in smaller Q82::GFP aggregates [17]. Another possible explanation for the seemingly different mobilities seen between the FRAP and FLIP studies of the mCherry::Ub protein within the aggregate is that the aggregates have subcompartments or are formed from multiple smaller aggregates. If this is the case, then it could be expected that bleaching an area of the aggregate will result in a quick recovery of fluorescence in the bleach area (as in the FRAP results), without reducing fluorescence in a separate part of the aggregate by exchanging mobile protein.

## Conclusion

These studies provide insight into the role of ubiquitination in a nematode model of polyglutamine protein aggregation. Time-lapse analyses of aggregate formation indicate an initial phase of growth that is likely spontaneous and not directly dependent upon ubiquitin. RNAi of *ubc-22 *affects the initial aggregation rate by increasing intracellular levels of soluble Q82::GFP fusion protein. Secondary growth is slower and appears to be more affected by ubiquitination. There is a period during the fourth larval stage in which ubiquitin is maximally located to Q82::GFP aggregates. Ubiquitin appears to be mobile, but sequestered within aggregates. These results suggest a model in which polyglutamine proteins misfold and rapidly form small aggregates, which subsequently attract substrates that have been ubiquitinated by a variety of E2 and E3 enzymes. The development of therapies for diseases involving loss of protein quality control may benefit from understanding how ubiquitination is involved in the handling of misfolded, damaged, or aggregating proteins.

## Methods

### Worm strains and maintenance

The Q82::GFP strain was created by Satyal et al. [47] and subsequently converted to integrated strain UA4 [49]. The LN139 strain harboring an integrated plasmid expressing the mCherry::ubiquitin fusion protein in muscle cells was created via microparticle bombardment with a plasmid created using the multisite Gateway system (Invitrogen). Entry plasmids for the *unc-54 *promoter, the *unc-54 *3' UTR, and the destination vector, pCR319, were gifts from Chris Ritchie. The entry plasmid with the mCherry::ubiquitin open reading frame was created by fusing mCherry to the N-terminus of ubiquitin in the pDONR221 vector. The LN149 strain (Q82 + Ub) was created by crossing the UA4 strain with LN139.

The temperature sensitive *uba-1 *strain (RV110) was obtained from Harold Smith and carries the *it129 *allele of *uba-1 *[50]. Males of this strain were crossed with the UA4 strain. A line showing 100% lethality at the non-permissive temperature and stably expressing the Q82::GFP transgene in the body wall muscle cells was propagated and used for the current study (LN150). Aggregates in the LN150 strain do not begin forming in significant numbers until approximately 24 hours after egg laying.

Worm strains were maintained according to standard methods [68]. Briefly, worms were cultured on nematode growth medium with *E. coli *strain OP50 and stored at 20°C, 16°C, or 25°C and 50 percent humidity. Worms for experimentation were chunked repeatedly from a stock plate containing starved worms. OP50 and HT115 *E. coli *strains were obtained from the *Caenorhabditis *Genetics Center.

### Nematode RNAi

RNAi feeding clones and procedures were previously described [17]. Petri plates containing 4.0 mL of NGM agar were supplemented with IPTG (Isopropyl β-D-1-thiogalactopyranoside) and ampicillin to achieve concentrations of 1 mM and 0.1 mg/mL, respectively. Plates were seeded with 125 μL of overnight HT115 culture and allowed to dry. L2 larval worms were transferred to RNAi plates and allowed to mature and lay eggs. L1 and L2 progeny were used for time-lapse observations. For RNAi treatment of Q82 + Ub worms in time course experiments, NGM plates containing 0.5% lactose and 100 mg/mL ampicillin were used to induce dsRNA production in HT115 bacteria.

### Microscopy and Time-lapse Analysis

Worms were washed from RNAi plates using M9 buffer and gravity sedimented for 5 minutes to pellet adults. Supernatants containing early larvae were pelleted by further gravity sedimentation (20 minutes) and washed 3X in M9 buffer to remove bacteria. Larvae were collected in a 15 μL drop of M9 and transferred to the center of a dried agarose pad on a glass microscope slide. Slides were mounted by applying a thin ring of petroleum jelly and placing a #1 coverslip over the ring.

Worms were illuminated continuously using a Nikon E600 epifluorescence microscope equipped with a 100-watt mercury lamp. A Nikon 10X objective was used in conjunction with the 2X optivar (200X total magnification). Neutral density filters (#16 and #8) were placed in the light path to reduce illumination intensity. Images were captured continuously (1 minute per exposure) using a Qicam 1394 Cooled-CCD monochrome camera, set at a gain of 0.6. Image Pro Plus 6.1 (Media Cybernetics) software was used to capture and process images.

Newly-formed aggregates were manually identified in the time-lapse imaging series. The total pixel intensity (on a scale of 0 to 4095) was measured over time in square regions of interest where aggregates appeared. Only singular, laterally stationary aggregates forming in L1 and L2 worms were recorded. Aggregates were imaged for 240 minutes. Data presented in Figure 3 begins at 30 minutes after the start of the observation period.

For time-lapse imaging of Q82::GFP in *uba-1 *mutants, worms were grown at the permissive temperature, 16°C. When plates were enriched with laid eggs, larvae and adult worms were washed from the plates, leaving only eggs. Plates with eggs were shifted to the non-permissive 25°C temperature. After 24 hours, hatched larvae were washed from plates and subjected to time-lapse microscopy as described above.

To observe secondary growth of preformed aggregates, the aggregates existing at the beginning of the time-lapse observation period were measured over time. The Track Objects feature in Image Pro Plus 6.1 was used to automatically find and measure the intensity of these objects. Aggregates overlapping in the Z axis or in close proximity to other aggregates were omitted.

To measure mCherry::ubiquitin colocalization to Q82::GFP aggregates, images were taken using a 40X lens, at multiple Z planes first for mCherry, then for GFP. Z-stacks were pseudocolored and merged to form 48-bit color z-stacks. The line profile measurement tool in Image Pro 6.1 was used to measure GFP and mCherry fluorescence across the center diameter of Q82::GFP aggregates

### Photobleaching experiments

All photobleaching experiments were performed using a Zeiss LSM710 laser scanning confocal system coupled to a Zeiss AxioObserver equipped with a 40X 1.2NA water-immersion lens. LN149 worms were chunked from stock plates and incubated for 72-hours. L4-stage and adult worms were mounted on slides in M9 containing 5 mg/mL tetramisole as described for time-lapse analysis. For FRAP, aggregates of interest in adult worms were viewed at a zoom level of 22.0, with a frame size of 51.8 μm^2^. Fluorescence was bleached to approximately 50% of initial intensity. Images were acquired every 100 ms using bidirectional scanning with a 555 nm laser (for mCherry) or 488 nm laser (for GFP) set to 0.5% power. 100 pre-bleach images were acquired prior to bleaching. Bleaching was directed to an area near the periphery of the Q82::GFP aggregates in a rectangular area of 0.79 μm^2 ^using the 555 nm laser at 100% power for 3 iterations or the 488 nm laser at 100% power for 1 iteration. A second, non-bleached region measured within the same aggregate was used to control for acquisition photobleaching. Mobile fraction (M_f_) was calculated from FRAP data using the equation M_f _= (I_f_-I_1_)/(I_0_-I_1_), where I_f _is the final fluorescence intensity in the bleached region after recovery, I_1 _is the mean fluorescence intensity immediately after bleaching, and I_0 _is the fluorescence intensity prior to bleaching.

For FLIP, worms were viewed at a zoom level of 8.0, with a frame size of 396.0 μm^2^. Images were acquired every 3 seconds using unidirectional scanning. After 6 prebleach images were acquired, repeated bleaching was directed at a square area of 0.81 μm^2 ^every 7 seconds using a 555 nm laser set to 100% and running for 10 iterations per bleach. Separate experiments were performed in which bleaches were directed to regions within the aggregate or the cytoplasm, and fluorescence measurements were collected for non-bleached regions within both the aggregate and the cytoplasm. To control for acquisition photobleaching, fluorescence was measured in separate cell that was not subject to FLIP. Relative fluorescence intensity was calculated using the following formula (I_t_/N_t_)/(I_0_/N_0_), where I_t _is the mean fluorescence intensity in the region of interest at a given time point, N_t _is the fluorescence intensity at non-bleached control region at a given time point, I_0 _is the initial (pre-bleach) mean fluorescence intensity in the region of interest, and N_0 _is the initial mean fluorescence intensity in a non-bleached control region.

### Immunofluorescence

Gravid Q82::GFP worms were age synchronized by treating gravid adults with hypochlorite solution to extract embryos. These progeny were allowed to hatch on a standard NGM plate and grow for 72 hours. Adult worms were then washed from the plates, washed 3X in M9 buffer, and once in distilled water. Concentrated worms were placed on a polylysine-coated slide and frozen in liquid nitrogen. The coverslip was removed and worms were fixed on the slides using -20°C methanol for 20 minutes. After several washes in PBS, slides were blocked for 30 minutes using normal goat serum. Worms were then incubated for 2 hours with the primary antibody (diluted 1:200) specific for K48 (Millipore clone Apu2) or K63-linked (Millipore clone Apu3) polyubiquitin chains. The secondary antibody (diluted 1:200) was rhodamine-conjugated goat anti-rabbit IgG (Jackson ImmunoResearch). Worms were imaged using a 40X lens. Images were collected at multiple Z-planes first for TRITC, then for Q82::GFP. Images were generated by flattening Z-stacks into composite extended depth-of-field images, which were pseudocolored and merged to view colocalization.

## Authors' contributions

GS performed the experiments and wrote the manuscript. LB created the transgenic mCherry::ubiquitin strain, provided oversight for the project, and edited the manuscript. All authors read and approved the final manuscript

## Supplementary Material

Additional file 1 **Video 1 This video shows a transgenic *C. elegans *larva expressing Q82::GFP fusion protein**. Several aggregates are formed during the course of filming. One hour of real time elapses in this video, and each frame represents 1 minute of real time. Brightness and contrast of the green-pseudocolored GFP fluorescence channel have been increased from the original images to show the diffuse, soluble material. A single DIC image is repeated for every frame and serves as an anatomical reference. The brightness and contrast have been adjusted for the DIC image to enhance visualization of the GFP channel. A 10X lens with a 2X optvar was used, to give 200X total magnification. Video plays at 10 frames/sec with each frame representing one minute of recording time. Click here for file

Additional file 2 **Video 2 This video shows aggregation prone fluorescent Q82::GFP fusion protein expressed in the body wall muscle cells of a transgenic *C. elegans *larva**. An aggregate is seen forming in the lower right of the image. The video represents 40 minutes of real time. Video plays at 10 frames/sec, with each frame representing one minute of recording time. A 10X lens with a 2X optvar was used, to give 200X total magnification. Brightness and contrast were increased to better show the diffuse, soluble material. Scale bar indicates a distance of 10 microns. Click here for file
